# Creation of Electron-doping Liquid Water with Reduced Hydrogen Bonds

**DOI:** 10.1038/srep22166

**Published:** 2016-02-26

**Authors:** Hsiao-Chien Chen, Fu-Der Mai, Bing-Joe Hwang, Ming-Jer Lee, Ching-Hsiang Chen, Shwu-Huey Wang, Hui-Yen Tsai, Chih-Ping Yang, Yu-Chuan Liu

**Affiliations:** 1 Department of Biochemistry and Molecular Cell Biology, School of Medicine, College of Medicine, Taipei Medical University No. 250, Wuxing St., Taipei 11031, Taiwan; 2 Department of Chemical Engineering, National Taiwan University of Science and Technology, No. 43, Sec. 4, Keelung Rd., Taipei 10607, Taiwan; 3 Graduate Institute of Applied Science and Technology, National Taiwan University of Science and Technology, 43 Keelung Rd., Sec. 4, Taipei 10607, Taiwan; 4 Core Facility Center, Office of Research and Development, Taipei Medical University, No. 250, Wuxing St., Taipei 11031, Taiwan; 5 Graduate Institute of Medical Science, College of Medicine, Taipei Medical University, No. 250, Wuxing St., Taipei, Taiwan

## Abstract

The strength of hydrogen bond (HB) decides water’s property and activity. Here we propose the mechanisms on creation and persistence of innovatively prepared liquid water, which is treated by Au nanoparticles (AuNPs) under resonant illumination of green-light emitting diode (LED) to create Au NP-treated (sAuNT) water, with weak HB at room temperature. Hot electron transfer on resonantly illuminated AuNPs, which is confirmed from Au L_III_-edge X-ray absorption near edge structure (XANES) spectra, is responsible for the creation of negatively charged sAuNT water with the incorporated energy-reduced hot electron. This unique electronic feature makes it stable at least for one week. Compared to deionized (DI) water, the resulting sAuNT water exhibits many distinct properties at room temperature. Examples are its higher activity revealed from its higher vapor pressure and lower specific heat. Furthermore, Mpemba effect can be successfully explained by our purposed hypothesis based on sAuNT water-derived idea of water energy and HB.

A unique feature of liquid water is its highly labile state of a network of hydrogen bonds (HBs), in which the breaking and reforming of HBs occur at a picosecond time scale^1^,^2^. HBs of liquid water have the dual functions of acting on itself to form water clusters and interacting with other species. The strength of acting on itself determines the size of water clusters; while the availability of interacting with other species is relative to the activity of liquid water. Theoretically, water deviates from the tetrahedral symmetry observed in bulk water, creating disordered defects that reduce the scale of water clusters. Compared to bulk bound water clusters, disordered water clusters with weak HBs have more free water molecules which can interact with other species to enhance the activity.

Because the O–H stretching frequency of water is very sensitive to environmental vibrations of spectroscopy, Raman scattering^3^,^4^ and infrared (IR) absorption^5^ at the same time scale of dynamic water are suitable for examining water’s HB structures. In addition, the difussionally averaged structure of local clusters of water can be evaluated by nuclear magnetic resonance (NMR). Liquid water is typically considered to be a bystander in chemical reactions. Recently, liquid water-based catalysts were reported for probing water micro-solvation of proteins by water-catalyzed proton-transfer tautomerism^6^ and photobiocatalytic chemistry of oxidoreductases using water as the electron donor^7^. In the water-gas shift reaction, gold (Au(111)) and Au nanoparticles (AuNPs) have a poor catalytic performance due to their inability to activate one of the most important steps of the reaction, the breaking of O–H bonds in the dissociation of water (H_2_O → OH + H)^8^. Velasco-Velez *et al.* reported that the interfacial water molecules at the Au electrode have a different structure from those in the bulk revealed from x-ray absorption spectroscopy (XAS) of electron yield^9^. Pang *et al.* reported that the electron bound in the intermediate of electron-hydronium ion-pair (EHIP) on the Ag and Au cathodes can be excited under light irradiation^10^. We supposed the relatively large energetic barrier can be overcome by utilizing resonantly illuminated AuNPs to facilitate the dissociation of H_2_O. This idea was derived from our innovatively prepared Au nanoparticle-treated (sAuNT) water under resonant illumination of green light emitting diode (LED) at room temperature^11^. Surface plasmon resonance (SPR) excited the illuminated AuNPs to decay into energetic hot electrons, and instantaneously, hot electron transfer (HET) was utilized to create sAuNT water with reduced HBs^11^. HET can promote many chemical reactions, including the dissociation of hydrogen^12^ and activation of oxygen^13^. Here we present mechanisms of the sAuNT water. In addition, some distinct properties, including the activity and low specific heat, of sAuNT water are described for the first time.

## Results and Discussion

### Creation and persistence of sAuNT water with reduced HB

**Figure 1:**
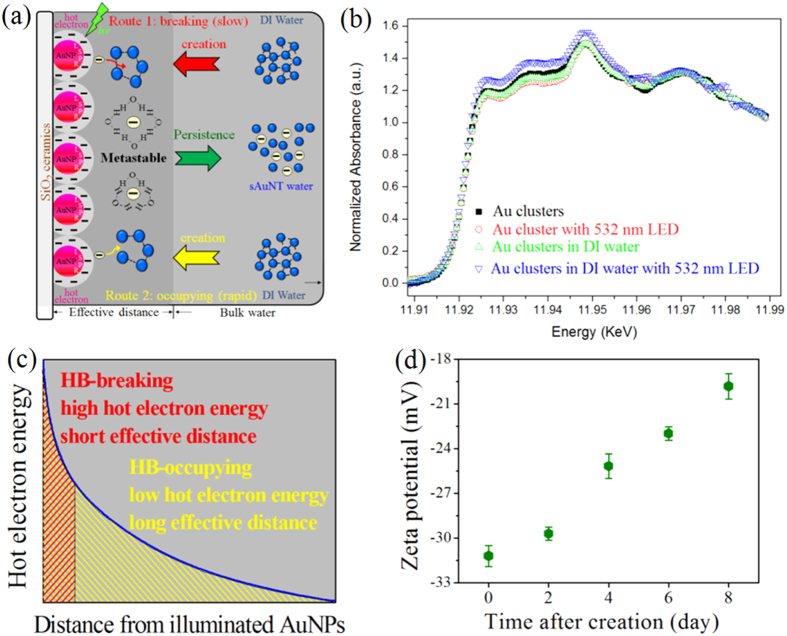
Mechanisms on creation and persistence of liquid water with reduced HB. **(a)** Schematic descriptions for the creation of sAuNT water. (**b**) Au L_III_-edge XANES spectra of Au clusters (solid black square), Au clusters with 532 nm LED (hollow red circle), Au clusters in DI water (hollow green up-side triangle), and Au clusters in DI water with 532 nm LED (hollow blue down-side triangle). (**c**) The schematic diagram showing the effective distance of hot electrons how to affect the HB in two distance domains. (**d**) Zeta potential of sAuNT water with respect to time after its creation.

Figure 1a shows a proposed mechanism for creating sAuNT water on SiO_2_ ceramic-supported AuNPs. Excited AuNPs decay non-radiatively into hot electrons with an energy level within the vacuum energy and Fermi level^14^, which reduces barriers for chemical reactions. The hot electron’s energy is critically dependent on the Au particle size and this energy is ca. 2.2~2.3 eV for particle sizes of 5~50 nm^12^. In addition, using photosensitive materials to support AuNPs loading creates additional channels within interfaces for electron transfer in an excited state, resulting in a reduction of available hot electrons^14^. In this study, AuNPs (~15 nm) were loaded on ceramic particles with ca. 92% dielectric support of SiO_2_. This dielectric support ensures the effective availability of produced hot electrons and localization of hot electron-related reactions taking place on the illuminated AuNPs surface. As bulk water diffuses and is adsorbed on illuminated AuNPs at an effective distance, hot electrons are captured by adsorbates of water molecules.

The evidence of captured hot electrons by water molecules is confirmed by Au L_III_-edge X-ray absorption near edge structure (XANES) spectra (Fig. 1b). XANES spectra is recorded, the first peak on the rising edge at 11.923 KeV results from the electronic transition from a 2p_3/2_ to the unoccupied “d” states near or above the Fermi level. This sharp peak is generally called as white line and its intensity is sensitive to the degree of electron occupancy in the valence orbits of the absorber^15^,^16^. Generally speaking, the lower the white line intensity, the higher the electron density and the lower the oxidation state of Au. As can be seen that the area of Au clusters, Au clusters with 532 nm LED, and Au clusters in H_2_O between 11.925 KeV to 11.95 KeV is almost the same, but that of the Au clusters in water with 532 nm LED is increase, indicating that the part amount of free electrons of the Au clusters is injected into the H_2_O and further increases the level of the unoccupied “d” states. It implies the water molecules would have excess electrons surrounded by its stereostructure.

The released hot electrons affect the water molecules through two paths. As proposed in route 1 of Fig. 1a, accumulating sufficient energy from HET to break H_2_O–H_2_O bonds (~23 kJ/mol) can be expected^17^. However, the reaction is time-consuming to accumulate, and it must occur in very close proximity to the illuminated AuNP surface. Besides, a faster process occurs during HB rearrangement. The dynamic processes of continuous and spontaneous forming, breaking, and rearranging of HBs are within picosecond scale which is accordant with the lifetime of hot electrons^18^. Therefore, hot electrons can seize the moment to rapidly occupy breaking HBs and prevent them from reforming. In addition, it was demonstrated that the energy of hot electrons is free path-dependent^14^, and their energy decreases with an increase in distance from the AuNP surface. For route 1, maintaining sufficient energy must be valid within several nanometers of the AuNP surface. On the contrary, the occupying route is spontaneous with no additional energy. Thus the effective distance of hot electrons can extend to tens of nanometers (Fig. 1c)^14^. The hot electrons cut the water network into “small fragments” forming sAuNT water and prevents them from recombining due to electrostatic repulsion. Free hot electrons can merge in sAuNT water by being surrounded by water molecules (interior-bound clusters) or being localized on the water cluster surface (surface-bound clusters)^19^. With the former mechanism, hydrogen atoms (with low electronegativity) are attracted to hot electrons, while oxygen atoms (with high electronegativity) are correspondingly exposed. This results in sAuNT water being slightly negatively charged. With the latter mechanism, water clusters are negatively charged because they are surrounded by hot electrons. The persistence of metastable sAuNT water is time-dependent, as suggested from the measured zeta potential of sAuNT water at −31.2 ± 0.70 (as prepared) to −19.8 ± 0.85 mV (after aging for 8 days) (Fig. 1d); while DI water was close to electronically neutral (−2.41 ± 0.31 mV). The prepared sAuNT water loses its activity with time due to destruction of the metastable conformation.

**Figure 2:**
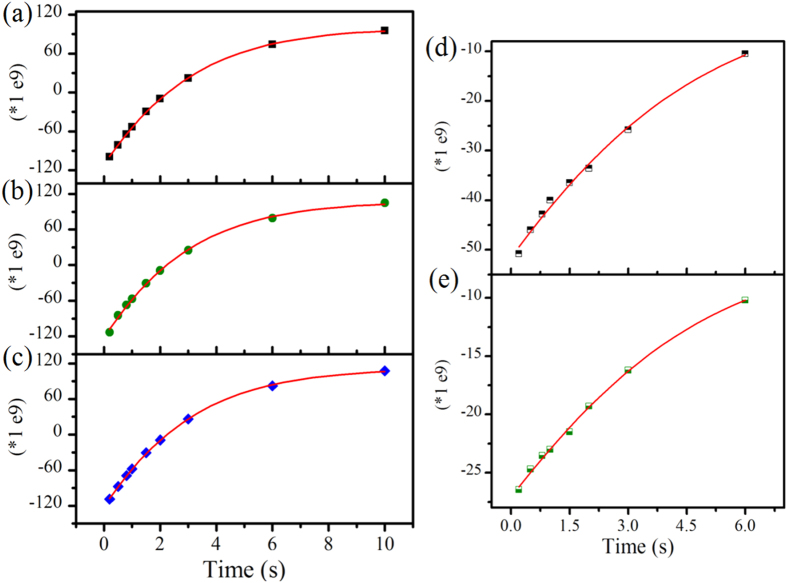
T_1_ represents the time required for the longitudinal component of magnetization to recover its equilibrium value after applying a perturbing pulse sequence. Spectra (**a–e**) represent plus of spectral signals as a function of repetition time for DI water, AuNT (light-free) water, sAuNT water, D_2_O, and sAuNT D_2_O.

The evidence for reduced HBs in sAuNT water was demonstrated by deconvoluted Raman spectra in O-H stretching vibrations (Figure S1) and the NMR relaxation time (T_1_) (Fig. 2). The degrees of non-HB structures (DNHBS) of water are 21.5 ± 0.06%, 24.5 ± 0.15% and 26.4 ± 0.19% for DI water, AuNT water (under fluorescent lamp illumination) and sAuNT water, respectively (see SI). Under magnetic field fluctuation, T_1_ of DI water is 3.092 s, shorter than 3.169 s of sAuNT water. Meanwhile, the T_1_ of 3.087 s for AuNT water (light-free) was very close to the value of DI water. This again demonstrates that light illumination of supported AuNPs is necessary to reduce HBs. This phenomenon was also observed for D_2_O and sAuNT D_2_O (see SI). Also, as reported in the literatures^20^,^21^, electrons would be correspondingly responded in magnetic field. Therefore, we found that the duration of metastable electron-doping sAuNT water can be effectively prolonged in external magnetic field (see SI and Figure S2). These analyses of Raman spectra and NMR relaxation times suggested the intrinsic reduction of HB structures in sAuNT water.

The density of water is dependent on its structural arrangement and conformation through HB-forming water-chain structures. As parcels of HBs are broken, “free” and “small” segments fill in the free volume of bulk water, thus slightly increasing the intrinsic density from 0.99705 ± 0.000000 g cm^−3^ of DI water to 0.99707 ± 0.000006 g cm^−3^ of sAuNT water (*n* = 3, the average value is obtained form three duplicated samples, n represents quantity of sample). The water network is similar to the relationship between monomers and polymers, in that the conformation of polymers is limited by the covalent bonds and is random, resulting in a higher free volume and lower density (see SI). This result can be also explained by a spatial density function of water^22^.

### Density of sAuNT water and sAuNT water-ethanol solution

Furthermore, the interaction of sAuNT water with ethanol was investigated by measuring the density. Hydroxyl groups of water form strong HBs with ethanol. The arrangement of ethanol as constructed by HBs is hindered by alkyl groups and results in an expectedly high free volume. Thus the intrinsic low density of ethanol was ca. 0.789 g cm^−3^. When 10% DI water was present in ethanol, the solution density significantly increased from a composition-averaged calculated value of 0.80981 g cm^−3^ to a measured value of 0.82057 ± 0.000015 g cm^−3^. This suggested that water clusters can fill in the free volume to enhance the packing effect. In addition, newly formed HBs substitute for original interactions among ethanol molecules and destroy the rigid order, thus decreasing the free volume and increasing the density. The density further increases to 0.82441 ± 0.000021 g cm^−3^ while the solution is treated by illuminated AuNPs, meanwhile ethanol is not decomposed during this treatment^23^. This result suggested that the distinct capability of providing more opportunities to form HBs with ethanol was indeed observed in sAuNT water (see SI).

### Activity of sAuNT water and its reductive activity

Water’s energy is associated with the bounded state of water molecules. It was reported matrix and osmotic effects^24^, involving HBs, capillary forces, and a requirement to restore water’s free state, reduces the potential (or activity) of water. This discourse is based on the assumption of “free water”, in which HBs do not exist. So far, changes in water’s activity in different states have not been revealed for pure water. Generally, the potential of pure water can be calculated according to the following equation^25^:


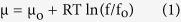


where μ and μ_0_ (J mol^−1^) are defined as the potentials (i.e., thermodynamic activities or energies); R is the gas constant (8.314 J mol^−1^ K^−1^); T is the temperature in K; and f and f_0_ are the respective vapor pressures of AuNT and DI waters. The vapor pressures of DI and AuNT waters are 0.0208 and 0.0327 bar at 298 K^11^. Compared to DI water, the activity of AuNT water was higher 1121 J mol^−1^. This suggests AuNT water possesses higher activity, which is in accordance with the increase in activity observed for confined water^26^. Based on the bonding energy of HBs (~23 kJ mol^−1^), it is thought the increased activity utilized in breaking HBs is equivalent to ca. 4.9%. This means that the increased non-hydrogen-bonded structure of 9.8% is formed in AuNT water. This value of 9.8% is basically in agreement with the increased DNHBS of 14.0% for AuNT water derived from the analysis of Raman spectra. This increased DNHBS of 14.0% for AuNT water is calculated from (DNHBS of 24.5% for AuNT water minus DNHBS of 21.5% for DI water) divided by DNHBS of 21.5% for DI water.

Moreover, the high activity of sAuNT water was examined by its ability to reduce AgNO_3_ with the aid of sodium citrate. The colorless sAuNT water-based AgNO_3_ solution turned light yellow after 1 h in darkness at 80 °C (Figure S3a and insert). However, this change was not observed in the DI water-based AgNO_3_ solution. It revealed sAuNT water accelerates Ag reduction, suggesting sAuNT water possesses higher potential energy to allow a lowering of the energy gap in the reduction of AgNO_3_ (see SI).

Furthermore, the activity of sAuNT water was examined by treating oxidized polyaniline (PAn). The band intensity corresponding to polaron decreased, accompanied by a blue shift of bipolaron^27^ when oxidized PAn was immersed in DI water (Figure S3b), suggesting the *p*-doping type of PAn can be slightly reduced due to washing out the dopant. These phenomena were much more significant to sAuNT water, indicating sAuNT water washed out dopant as well as provided electrons, which remain in a metastable state, to reduce the *p*-doping type of PAn.

### Heat capacity of sAuNT water

**Figure 3:**
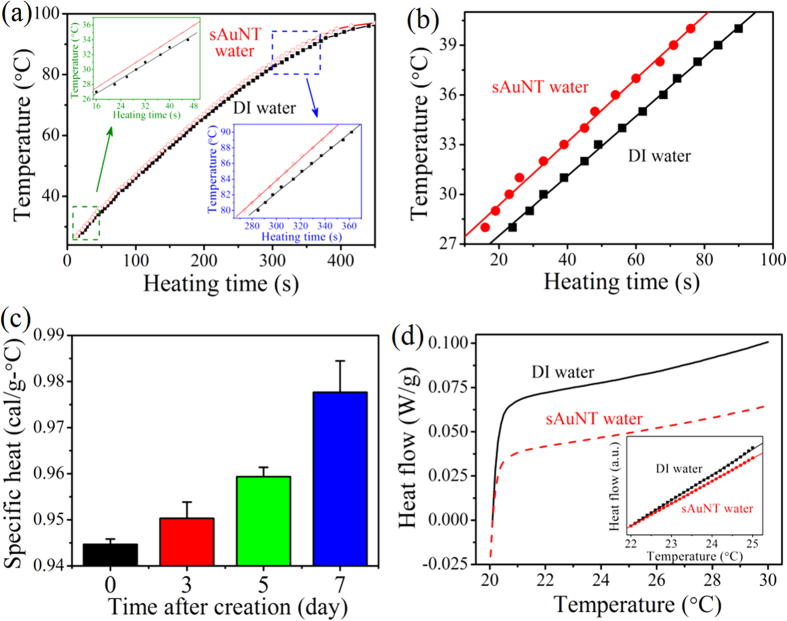
Specific heat of sAuNT water compared to DI water. (**a**) Rates of rising temperatures measured in DI and sAuNT waters with the same masses under a constant applied power. (**b**) The temperature-heating time dependence of DI and sAuNT waters. (**c**) The specific heat of sAuNT water was time-dependent after its creation. (**d**) DSC measurements of DI and sAuNT waters.

Calculating the activity also disclosed a difference in vapor pressure which indicated the different heat capacity. As expected, temperatures of both waters increased with the heating time (Fig. 3a). At temperatures above 90 °C, lines became flattened due to nearly boiling. The boiling point was ca. 97.3 °C for DI water; while it was reduced to ca. 94.1 °C for sAuNT water. It is recognized that HBs serve as a storeroom of energy. A portion of the heat is used to break HBs rather than to directly raise temperature. Hence, the heat capacity should decrease with an increasing temperature because fewer HBs remain at higher temperatures. Contrarily, it increased with rising temperatures. This reveals the added heat is used to break HBs and raise the temperature, and especially, to maintain the degree of freedom of water molecules to prevent them from re-bonding. Therefore, sAuNT water possesses intrinsically fewer HBs and can prevent the re-bonding of HBs, thus reducing the energy gap with a rising temperature. The specific heat of sAuNT water measured between 25 and 40 °C demonstrated a reduced value of 0.945 ± 0.0012 cal g^−1^ °C^−1^ (*n* = 3) in contrast to the 1.000 cal g^−1^ °C^−1^ of DI water (Fig. 3b). To a general difference in specific heats of < 1% for DI water^28^ at low and high temperatures, the 6% difference was significant. In addition, sAuNT water transformed into DI water with time, resulting in increasing specific heat (Fig. 3c). This result was consistent with the result of zeta potential (Fig. 1d) (see SI). The heat capacity was also measured by differential scanning calorimetry (DSC) (Fig. 3d). The measured heat capacity of sAuNT water between 22 and 25 °C was ca. 0.896 cal g^−1^ °C^−1^ in contrast to the 1.000 cal g^−1^ °C^−1^ of DI water. In addition, the difference in the heat flow between sAuNT and DI waters became clearer at higher temperatures. These results are in agreement with the correlation of the water cluster size and heat capacity, in which the heat capacity of (H_2_O)_21_ was smaller than that of (H_2_O)_50_ at 27 °C^29^.

### Explanation of Mpemba effect based on sAuNT water

**Figure 4:**
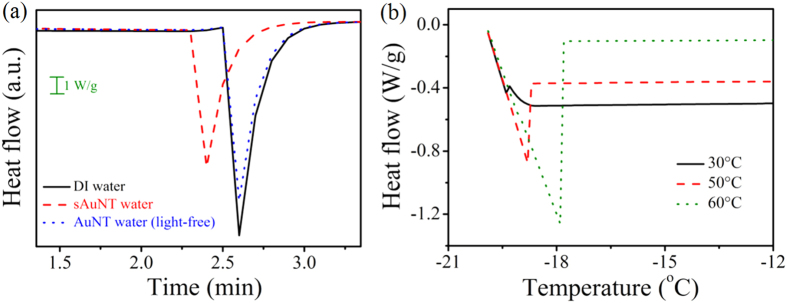
Explanation of the Mpemba effect. (**a**) The thermodynamic responses of DI water, AuNT water (light-free), and sAuNT water under the same cooling rate of 30 °C/min. (**b**) The thermodynamic responses of DI water with different initial temperatures under the same cooling rate of 30 °C min^−1^.

Furthermore, the freezing speeds of waters were measured by DSC (Fig. 4a). Initially, water does not freeze below 0 °C, meaning the supercooled liquid water is not in thermodynamic equilibrium^30^. It was observed that the freezing time for sAuNT water was 2.4 min which was faster than that of 2.6 min for DI water. Also, the time of AuNT water (light-free) was the same to DI water. In the freezing stage, the first general requirement for freezing of liquid water is the presence of a nucleus which is constructed by water molecules in an arrangement with a well- defined order^31^. Then, free water molecules in the vicinity of the nucleus are attracted to the nucleus for further crystallization. Finally, the growths of nuclei and crystalline structures result in the freezing^31^. Nevertheless, adequate energies are necessary during the freezing processes for ordering and attracting. A portion of water characteristics, in which molecules are connected by HBs and covalent bonds, are similar to those of polymers. Extending or heating polymers helps arrange or crystallize polymer chains^32^. Hence, providing additional energy is favorable for freezing water. It was previously stated that sAuNT water with fewer HBs possesses higher energy potential. Stored energy is available for water molecules with extra kinetic energy when the ambient temperature rapidly decreases. Namely, the non-HB structure in a high-energy state can transform into an HB structure in a low energy state, accompanied by a release of available energy. This innovative phenomenon may be used to explain the occurrence of the Mpemba effect^33^, in which warm water freezes more quickly than cold water. This issue is still an interesting mystery in physics to the present. Various explanations based on evaporation^34^ and convection^35^ have been investigated, but the phenomenon is not yet completely understood. So far, there has been less discussion of the Mpemba effect from the concept of the inherent energy of water molecules. As shown in Fig. 4b, the Mpemba effect was observed by comparing the freezing times of DI waters with different temperatures. It is known that the degree of HBs in water molecules is dependent on the temperature. HBs in water can be broken by raising the temperature. As warm water is freezing, the broken HBs revert again and can release their initially stored energy for easy ordering, thus freezing quickly. Therefore, we propose a new explanation of the Mpemba effect that the reduced degree of HB structure observed in warm bulk water or sAuNT water at room temperature. Mindfully, the contribution of impurity to sAuNT water for exhibiting distinct properties could be excluded (see SI).

In summary, we innovatively utilized the LSPR of AuNPs to reduce HBs of water *via* breaking and occupying processes by hot electrons. The corresponding mechanisms of the creation and persistence of sAuNT water are successfully explained by experimental evidence. Compared to bulk DI water, this sAuNT water possesses many novel properties, including a lower specific heat, which are demonstrated for the first time herein. In addition, we propose a new hypothesis, which can explain the mysterious puzzle of the Mpemba effect. We believe these new findings will lead to new ideas for potential applications in medicine, biology, and chemistry based on water with a reduced HB structure.

## Methods

### Chemicals and Materials

Electrolytes of NaCl (>99%), dehydrate ethanol, aniline (99.5%) and KCl (>99%) were purchased from Sigma-Aldrich Organics. Reagents of sodium hydroxide (NaOH, 98.5%) and AgNO_3_ (99.85%) were purchased from Acros Organics. Sulfuric acid (>95%) sodium citrate (99.9%) were purchased from Fisher. HCl (37%) was obtained from Nihon Shiyaku Reagent. All of the reagents were used as received without further purification. 40 mesh-screened ceramic particles (Molar compositions: 92% SiO_2_, 3.0% Na_2_O and K_2_O, 2.0% Fe_2_O_3_, 1.5% Al_2_O_3_, 0.5% CaO, 0.5% MgO, and other rare metal oxides) for filtering through drink water were purchased from Chyuan-Bang enterprise Co., Ltd., Taiwan. Commercial chitosan (Ch) powders with a degree of deacetylation of 0.82 were purchased from First Chemical Works, Taiwan. All of the solutions were prepared using deionized (DI) 18.2 MΩ cm water provided from a Milli-Q system. All of the experiments were performed in an air conditioned-room at ca. 24 ^o^C. The water temperature is ca. 23.5 ^o^C.

### Preparation of Gold Nanoparticles

The AuNPs in an aqueous solution was obtained from an Au sheet (purity of 0.9999) by using electrochemical and thermal reduction methods, as shown in our previous report^36^. Typically, the Au electrode was cycled in a deoxygenated aqueous solution of 40 mL containing 0.1 M NaCl and 1 g L^−1^ Ch from −0.28 to + 1.22 V vs Ag/AgCl at 500 mV s^−1^ for 200 scans under slight stirring. The durations at the cathodic and anodic vertices are 10 and 5 s, respectively. Immediately, without changing the electrolytes, the solution was heated from room temperature to boiling at a heating rate of 6 ^o^C min^−1^ in air. After cooling the clear Au NPs-containing solution was separated from the settlement of Ch. Then the AuNPs-containing solution was placed in an ultrasonic bath for 30 min and was further centrifugalized at 3600 rpm for 2 min to remove Ch for preparing pure AuNPs in solution.

### Preparation of Cramic Prticles-supported Au NPs

The rinsed ceramic particles were immersed in a solution containing 30 ppm AuNPs for 1 day. Then the Au NPs-adsorbed ceramic particles were rinsed throughout with DI water, and finally dried in an oven at 100 ^o^C for 1 day. Subsequently, the prepared AuNPs-adsorbed ceramic particles were loaded in a valve-equipped glass tube (I.D.: 30 mm, L: 300 mm). Before prepare the sAuNT water the ceramic particles-supported AuNPs in the glass tube were rinsed with DI water for several cycles until the pH values of DI water before and after it passed through the particle-loaded tube are almost identical (ca. pH 7.23 and water temp. at ca. 23.5 ^o^C).

### Preparation conditions of AuNT water, AuNT water (light-free) and sAuNT water

In preparations of AuNT water, DI water (pH 7.23, T = 23.5 ^o^C) flew through the glass tube filled with AuNPs-adsorbed ceramic particles under fluorescent lamp illumination. Then the AuNT water (pH 7.25, T = 23.3 ^o^C) was collected in glass sample bottles for subsequent tests as soon as possible. For examining the purity of the prepared AuNT water further ICP-MS analyses indicated that the concentrations of the slightly dissolved metals in the AuNT water are ca. 0.62, 43, 25, 23, 13, 4.5 and 0.41 ppb for Au, Na, K, Al, Mg, Ca and Fe, respectively. Excluding Au, the total equivalent molar concentration of these dissolved metals is equal to ca. 6.9 × 10^−6^ N. This measured value is ca. 2.4 × 10^−7^ N for DI water as a reference. Similarly, the AuNT water (light-free) is produced according to the above processes in dark room without illumination. sAuNT water is also produced according to the above processed but under green light LED (532 nm) illumination.

### Measurement of zeta potential on water

Zeta potentials of water samples (600 μL) were analyzed using a Malvern Zetasizer Nano ZS zeta potential analyzer. To avoid the influence of bacteria on zeta potential during storage, samples were filtered by using membrane with 0.22 μm pore-size to remove bacteria. Filtered water was divided into 15 parts (n = 3 for each measurement). The parafilm-sealed samples were placed in dark atmosphere before measurement.

### Raman spectra recorded on water and their deconvolutions

In measurement prepared water (0.1 mL) was added into a cell with Ag sheet at the bottom of the cell. Raman spectra were obtained (Micro Raman spectrometer, Model UniRAM-Raman) by using a confocal microscope employing a diode laser operating at 532 nm with an output power of 1 mW on the sample. A 50×, 0.36 NA Olympus objective (with a working distance of 10 mm) was used to focus the laser light on the sample-containing Ag sheet. The laser spot size is ca. 2.5 μm. A thermoelectrically cooled Andor iDus charge-coupled device (CCD) 1024 × 128 pixels operating at −40 ^o^C was used as the detector with 1 cm^−1^ resolution. All spectra were calibrated with respect to silicon wafer at 520 cm^−1^. In measurements, a 90^o^ geometry was used to collect the scattered radiation. A 325 notch filter was used to filter the excitation line from the collected light. The acquisition time for each measurement was 1 s. Thirty sequential measurements were collected for each sample.

### NMR relaxation time measurement

The NMR-T_1_ of DI water, sAuNT water and D_2_O were measured at atmospheric pressure by using a Bruker Fourier 300 spectrometer, operating at the 300 MHz ^1^H resonance frequency. In measurement, repetition time of 10 s and acquiring eight signal averages were employed for each delay time. The data was analyzed with the help of Bruker T_1_ analytical routine with a software package.

### Density measurement

Densities were measured with an Anton Paar DMA-4500 vibrating-tube densitometer, Austria^37^, having uncertainty of ±5 × 10^−5^ g.cm^−3^. The densitometer is equipped with an internal temperature control unit, which can regulate the temperature of the measuring tube to within ±0.03 K in a temperature range of (273.15 to 363.15) K. For this kind of instrument, the sample fills a vibrating tube. Usually, it is assumed that the densitometer behaves as an oscillator without damping. This is true for low-viscosity samples, but for high-viscosity ones the damping contribution is significant, a fact that makes the “measured” density higher than the real one^38^. Therefore, corrections for high-viscosity samples are needed for this kind of densitometer. There are some studies that deal with this problem, in which different procedures are proposed to make such corrections^39^. All of these methods require knowledge of the viscosity of the sample in order to obtain the correction through a simple analytical expression. The main advantage of the DMA-4500 densitometer is its ability to carry out this correction automatically, without any prior information about the viscosity of the sample. The instrument was calibrated with air and degassed distilled water.

### Chemical activity of water in reduction preparation of AgNPs

Precursors of AgNO_3_ and weak reducing agents of sodium citrate were added in DI water or sAuNT water in a glass sample cell (20 mL). The final concentrations of AgNO_3_ and sodium citrate in solution are 100 ppm and 30 mM, respectively. Then the Al foil-covered glass cell was heated to 80 ^o^C and maintained at this temperature for 1 day in ambient laboratory air. The obtained AgNPs in solution was diluted to 1/4 of its original concentration before characterizing it plasmon absorption band by UV-vis absorption spectrum.

### Preparation of doped PAn and its treatment by water

The electrochemical polymerization of polyaniline (PAn) was performed in conventional three-electrode cell, in which indium tin oxide (ITO) electrode as the working electrode, Pt sheet as the counter electrode and Ag/AgCl as reference electrode. The reaction was based on 0.5 M H_2_SO_4_ containing 0.1 M aniline. The electrochemical polymerization process was performed by potential cycling from −0.2 to 1.0 V vs Ag/AgCl at a scanning rate of 100 mV s^−1^ for 20 cycles. Then the film on the ITO was washed by DI water and 0.1 M NaOH to obtain a blue PAn film. After drying at room temperature for 1 day, PAn was further oxidized by dipping the PAn-deposited ITO into 0.1 M HCl to form a doping type PAn with green color. Also, after this drying process, it was dipping into DI water or sAuNT water for 15 min. The doping level (or oxidation level) was analyzed by absorption spectrum through Perkin-Elmer Lambda 800/900 spectrophotometer.

### Heating process on water for obtaining its specific heat

100 g DI water (or sAuNT water) was added in a glass beaker (250 mL). Then the opening of the glass beaker was covered with wrap and the water-containing glass beaker was heated from room temperature to boiling point by using a heater (Corning, pc-420D) in ambient laboratory air. The temperature of water was recorded by using a temperature meter (First clean corporation, pH 500). The mass losses at 40 ^o^C and boiling point are 0.13 (or 0.43) and 7.94 (or 9.15) g for DI water (or for sAuNT water), respectively.

### DSC measurement

The thermodynamic properties of DI water and sAuNT water were studied by differential scanning calorimetry (DSC; TA Q20). In measurement on heat capacity, 10 mg water samples in DSC holders were heated from room temperature at 1.5 °C min^−1^ under nitrogen gas at a flow rate of 10 mL min ^−1^. To reduce the experimental error from few mass of sample the heat capacity of sAuNT water referred to known heat capacity of known bulk DI water was calculated in the temperature ranging from 22 to 25 °C with well linear heat flow-temperature dependence. In cooling experiment, 10 mg water samples in DSC holders were cooled from room temperature at 30 °C min^−1^ under nitrogen gas at a flow rate of 10 mL min ^−1^. The temperature at −20 °C was kept for 10 min before terminating experiment.

## Additional Information

**How to cite this article**: Chen, H.-C. *et al.* Creation of Electron-doping Liquid Water with Reduced Hydrogen Bonds. *Sci. Rep.* **6**, 22166; doi: 10.1038/srep22166 (2016).

## Supplementary Material

Supplementary Information
