# Supplementary material for: Creation of Electron-doping Liquid Water with Reduced Hydrogen Bonds
Source: Sci Rep. 2016 Feb 26;6:22166. doi: 10.1038/srep22166 (PMC4768145; doi:10.1038/srep22166)
Supplement: Supplementary Information [file srep22166-s1.doc]

**Supplementary Information**

**Creation of Electron-doping Liquid Water with Reduced Hydrogen Bonds**

Hsiao-Chien Chen^1^, Fu-Der Mai^1^, Bing-Joe Hwang^2^, Ming-Jer Lee^2^, Ching-Hsiang Chen^3^, Shwu-Huey Wang^4^, Hui-Yen Tsai^1^, Chih-Ping Yang^5^ & Yu-Chuan Liu^1^,*

^1^ Department of Biochemistry and Molecular Cell Biology, School of Medicine, College of Medicine, Taipei Medical University No. 250, Wuxing St., Taipei 11031, Taiwan. ^2^ Department of Chemical Engineering, National Taiwan University of Science and Technology, No. 43, Sec. 4, Keelung Rd., Taipei 10607, Taiwan. ^3^ Graduate Institute of Applied Science and Technology, National Taiwan University of Science and Technology, 43 Keelung Rd., Sec. 4, Taipei 10607, Taiwan. ^4^ Core Facility Center, Office of Research and Development, Taipei Medical University, No. 250, Wuxing St., Taipei 11031, Taiwan. ^5^ Graduate Institute of Medical Science, College of Medicine, Taipei Medical University, No. 250, Wuxing St., Taipei, Taiwan.

^*^ Corresponding author

Tel: 886-2-27361661 ext 3155; Fax: 886-2-27356689; E-mail: [liuyc@tmu.edu.tw](mailto:liuyc@tmu.edu.tw)

**Supplementary Discussion**

1. Raman spectra on water samples

Raman spectrum is valid to characterize the distributions of HB structures in water. It provides the essential features of inter- and intra-interactions in various regions including translational mode, librational mode, OH bending and stretching vibration^3,4^. Figures S1a, S1d and S1g show the Raman spectra of DI, AuNT and sAuNT waters (n=6) in the region of 2700 to 3900 cm^–1^ related to the OH stretching vibration. Almost the same shapes of vibration band for six individual DI water samples in different batches indicate that the characteristic of hydrogen bond examined by using Raman spectrum is feasible. By further deconvolution, the degrees of non-hydrogen-bonded structure (DNHBS) of water are 21.5±0.06 %, 24.5±0.15 % and 26.4±0.19 % for DI water, AuNT water and sAuNT water, respectively (Fig. S1b, S1e and S1h). These deconvoluted data of DNHBS are discriminating between these three examined water samples even under extreme fitting conditions. The extreme values of DNHBS for DI water, AuNT water and sAuNT water range from 20.01 to 22.7%, from 24.23 to 25.07% and from 25.91 to 27.07%, respectively (Fig. S1c, S1f and S1i), based on deliberately deviated fitting from optimal one.

2. NMR relaxation times on water samples

Proton NMR relaxation times have been used to probe the interactions of water molecules with their surroundings. Therefore, the sAuNT water with reduced hydrogen-bonded structure compared to bulk water is also confirmed by the spin-lattice relaxation time measured on a 300 MHz NMR instrument. Under magnetic field fluctuation, the relaxation time T_1_ of DI water is 3.092 s, shorter than 3.169 s of sAuNT water. Meanwhile, T_1_ of 3.087 s for AuNT water (light-free) is very closed to value of DI water (Fig. 2a-c). It demonstrates again that light illumination on supported AuNPs is necessary for the creation of treated water with reduced hydrogen-bonded structure. The relaxation time T_1_ of sAuNT water is 1.029± 0.0035 times longer than that of DI water (n=3). The longer relaxation time indicates the lower degree of hydrogen-bonded interaction between water molecules in sAuNT water, resulting in reducing the effective proton transfer between spins and lattice. The T_1_ of 3.018 s (n=2) for DI water solution containing 50 wt% of sAuNT water is between values of DI water and sAuNT water. It suggests that the DNHBS in water is tunable. In addition, the longer relaxation time of ca. 5.580 s is also observed on the sAuNT treated D_2_O (Fig. 2d-e), compared to untreated D_2_O (ca. 5.063 s). These analyses on Raman spectra and NMR relaxation time suggest the intrinsic reduction of hydrogen-bonded structure in sAuNT water after our proposed innovative process.

3. The effect of magnetic field to sAuNT water

As reported in the literature,^18,19^ electrons would be correspondingly responded in magnetic field. This fact encourages us to further examine the effects of external magnetic fields on the persistence of the created electron-doping. Figure S2 shows the corresponding spin-lattice relaxation time of DI water and sAuNT water in NMR experiments at 300 MHz. Figure S2a-e in the same batch experiments are demonstrated to examine the effect of magnetic field in NMR experiments, while Figure S2f-i in the another batch experiments are demonstrated to examine the effect of magnetic field induced by strong magnet. Similarly, as shown in Figure S2a and 1b for as-prepared waters, the relaxation time T_1_ of DI water is 3.098 ± 0.0185 s, shorter than 3.191 ± 0.0029 s of sAuNT water. The relaxation time T_1_ of sAuNT water is ca. 1.030 times longer than that of DI water. Interestingly, as comparing Figure S2d and S2e with S2c for 10-day-aged waters, it was found that the relaxation time T_1_ of DI water (the same water used in Figure S2a) is 3.053 ± 0.0137 s, still significantly shorter than 3.134 ± 0.0114 s of sAuNT water (the same water used in Figure S2b). For aged water samples performed NMR experiments under magnetic field fluctuation 10 days ago, the relaxation time T_1_ of sAuNT water is ca. 1.027 times longer than that of DI water. This increased time for aged waters is close to that of 1.030 times for as-prepared waters, suggesting that magnetic field fluctuation acting on as-prepared electron-doping sAuNT water may rearrange its metastable state to make it more stable. However, this magnetic field fluctuation influences less on the as-prepared DI water, making its hydrogen-bonded structure changed slightly. In addition, T_1_ of 3.058 ± 0.0153 s for aged sAuNT water (Figure S2d for sAuNT water without performing NMR experiment in its fresh state) is very closed to the value of aged DI water (Figure S2c). It indicates that the metastable state of sAuNT water with reduced hydrogen bonds, compared to DI water, would be completely destroyed and restored to the normal hydrogen-bonded structure of DI water after its creation for 10 days without additional magnetic field fluctuation. Again, we performed NMR experiments of DI water and sAuNT water. Similarly, as shown in Figure S2f and 1g for as-prepared waters, the relaxation time T_1_ of DI water is 2.989 ± 0.0021 s, shorter than 3.063 ± 0.0453 s of sAuNT water. The relaxation time T_1_ of sAuNT water is ca. 1.025 times longer than that of DI water. Encouragingly, as comparing Figure S2i with 1h for 14-day-aged sAuNT waters with and without placing on strong magnet during their storing, it was found that the relaxation time T_1_ of sAuNT water (placing on strong magnet) is 3.345 ± 0.0127 s, significantly longer than 3.170 ± 0.0658 s of sAuNT water (without placing on strong magnet). The relaxation time T_1_ of aged sAuNT water on strong magnet is ca. 1.055 times longer than that of aged sAuNT water far away from magnetic field. This result suggests that the duration of metastable electron-doping sAuNT water can be effectively prolonged in external magnetic field.

4. Densities of water and water-ethanol solution

The density of water is dependent on its structure arrangement and conformation forming with other neighbor water molecules through hydrogen bond connecting. The interaction linking within each molecule forms water-chain structure. As the parcel of hydrogen bonds are broken, the “free” and “small” segments will fill into the free volume in bulk water, thus slightly increase the intrinsic density from 0.99705±0.000000 g cm^–3^ of DI water to 0.99707±0.000006 g cm^–3^ of sAuNT water (n=3). Although the density does not change obviously, the increased value is reliable to the measurement for the tiny error. The water cluster resulted from hydrogen bonding is like the relationship between monomer and polymer, in which the conformation of polymer is limited by the covalent bonds and is random, resulting in higher free volume and lower density. In contrast, the highly packing monomer can reduce the free volume and thus increase its density because of less steric hindrance. This result can be also explained by a spatial density function of water^20^.

Furthermore, hydroxyl group in water can provide the major strong interaction of hydrogen bond with ethanol molecule. The arrangement of pure ethanol molecules by forming hydrogen bond is hindered by the presence of alkyl group. This loose arrangement due to the increase of steric hindrance results in an expected high free volume. Thus the intrinsic low density of ethanol is ca. 0.789 g cm^–3^. However, when 10 % DI water is present in ethanol, solution density significantly increases from a calculated value of 0.80981 g cm^–3^ based on densities and compositions of individual components to a measured value of 0.82057±0.000015 g cm^–3^. It suggests that water clusters can fill into the free volume of ethanol to enhance its packing effect. In addition, the formation of hydrogen bond between ethanol and water molecules substitutes for the original interaction between ethanol molecules. It destroys the rigid order, thus decreases the free volume and increases the corresponding density. This density can further increase to 0.82441±0.000021 g cm^–3^ while the solution is treated by resonantly illuminated AuNPs for changing the DI water into sAuNT water in solution. On the other hand, ethanol is not decomposed during this treatment^21^. It indicates that more free volume within ethanol is filled with sAuNT water and more original interaction between ethanol molecules is substituted by stronger hydrogen bond between ethanol and sAuNT water due to its small segments and more interaction sites available. The increase in density of sAuNT water -based ethanol solution results from its enhanced effects on closed-packing in free volumes of ethanol molecules and effectively shortening the distance of hydrogen bond. In addition, the densities of ethanol solutions (50 % water) based on DI water and sAuNT water are 0.92414±0.000030 g cm^–3^ and 0.92483±0.000020 g cm^–3^, respectively. Compared to ethanol solutions with 10 % water, the contribution from the sAuNT water to the increased density of ethanol solution with 50 % water is slight. These phenomena are reasonable because the available sites that can be substituted for hydrogen bonding structure between ethanol and water are sufficiently occupied by 50 % DI water at this highly water-containing solution. Compared to DI water, although sAuNT water is capable of forming more hydrogen bonds with ethanol this ability is suppressed at limited interaction sites available. This result suggests that a distinct capability of providing more opportunities on forming HB with ethanol is indeed observed on sAuNT water.

5. Reducing activity of sAuNT water on preparation of AgNPs

The high activity of sAuNT water is examined by its reducing ability on AgNO_3_ with the aid of sodium citrate. The colorless sAuNT water-based AgNO_3_ solution turns to light yellow after 1 hr in darkness at 80 ^o^C. However, this change is not observed in the DI water-based AgNO_3_ solution. This phenomenon reveals the sAuNT water can accelerate the Ag reduction. Both the sAuNT water-based and the DI water-based AgNO_3_ solutions turn to yellow after 24 h at 80 ^o^C. The UV-vis absorption spectrum shows a maximum band at 443 nm, which indicates the successful reduction of AgNO_3_ to Ag NPs (Figure S3a). Additionally, the wavelength of SPR is lower than that based on DI water, indicating that the prepared Ag NPs in sAuNT water are smaller and more concentrated. Generally, the aqueous solution should be boiled for the reduction of AgNO_3_ to Ag NPs by using reducing agent of sodium citrate. These results suggest that, compared to DI water, sAuNT water owns higher potential energy to allow lowering the energy gap in the reduction of AgNO_3_ to Ag NPs. Thus it can lower the reaction temperature and shorten the reaction time in reduction of AgNO_3_ by using sAuNT water.

6. Reducing activity of sAuNT water on oxidized PAn

The distinct activity of sAuNT water is examined by dipping of oxidized polyaniline (PAn) in it. In UV-vis absorption spectrum, PAn exhibits an absorption maximum band at around 625 nm, which originates from the π–π* transitions of quinoid exciton bands^26^. As dipped in acidic solution the band of the quinoid ring nearly disappears, which is due to the protonation of the imine groups of PAn. Meanwhile, the quinoid ring next to the protonated imine becomes a semiquinoid radical cation, which results in a decrease of the intensity of the exciton absorption band and generations of polaron and bipolaron absorption maximum bands at 435 and 880 nm, respectively, under dry state (Figure S3b) ^23^. The band intensity corresponded to polaron decreases, accompanying with a blue shift of bipolaron, when the oxidized PAn is immersed in DI water for 15 min. It suggests that the *p*-doping type of PAn can be slightly reduced due to effect of de-doping from DI water. However, these phenomena are much more significant in the sAuNT water system. It indicates that the sAuNT water not only can contribute de-doping effect but also can provide the electrons, which are kept in the metastable state of sAuNT water, to reduce the *p*-doping type of PAn, thus lowers the doping level of PAn further.

7. Heat capacities of sAuNT water

In the calculation of sAuNT water activity through the equation (1) it also discloses the difference of vapor pressure of sAuNT water from that of DI water at room temperature. It indicates that the heat capacity of sAuNT water would be different from that of DI water. Figure 3a shows the temperature corresponding to the heating time for 100 g of DI water and sAuNT water under constant heating power. As expected, the temperatures in both waters increase with the heating time, but the raising lines are not completely linear. As the temperature above 90 ^o^C, the lines become flatten, suggesting that these waters are nearly at boiling states. The boiling point is ca. 97.3 ^o^C for DI water; while this boiling point is reduced to ca. 94.1 ^o^C for sAuNT water. These results mean that sAuNT water is more ready to achieve the equilibrium state between its vapor pressure and atmospheric pressure. In heating on water, the provided energy can induce water molecular vibrations and rotations, thus, water’s apparent temperature is correspondingly raised. It is recognized that the hydrogen bond in water serves as a storeroom for energy. Therefore, partial energy from heating is used to break the hydrogen bonds in water rather than to directly raise its temperature. Based on this concept, the heat capacity of water should decrease with increasing temperature because of fewer hydrogen bonds remained at higher temperatures. Contrarily, as shown in Figure 3a, the slopes for both waters decrease with the rising temperature under supplying equal powers, meaning that specific heats of both waters increase with raising temperatures from 25 ^o^C to their boiling temperatures. It reveals the adding heat is used to break hydrogen bond and to raise temperature, especially, as well as to maintain the degrees of freedom of water molecules to prevent from the re-bonding between water molecules. Compared to DI water, network of sAuNT water owns intrinsically fewer hydrogen bonds. It can prevent it from re-bonding of hydrogen bonds, thus reduces the energy gap in raising temperature. Therefore, the rate in raising temperature for sAuNT water is faster than that for DI water. The measured specific heat of sAuNT water between 25 and 40 ^o^C demonstrates a reduced value of 0.945± 0.0012 cal g^-1^ ^o^C^-1^ (n=3) referred to 1.000 cal g^-1^ ^o^C^-1^ of the DI water (Figure 3b). Compared to a general difference in specific heats less than 1% for DI water^20^ at low and high temperatures, the 6% difference in specific heats between sAuNT water and DI water is significant. It exhibits again the effect of intrinsically reduced hydrogen bonds of sAuNT water on its distinct properties, which are quite different from those known on bulk water. Besides, the metastable sAuNT water transfers into DI water with time, resulting in the corresponding increase of its specific heat (Figure 3c). This result is consistent with the change of zeta potential of sAuNT water with time after its creation (Figure 1d)

The heat capacity of water was also measured through differential scanning calorimetry (DSC) at a heating rate of 1.5 ^o^C min^-1^ (Figure 3d). The measured heat capacity of sAuNT water between 22 and 25 ^o^C with well linear heat flow-temperature dependence also demonstrates a reduced value of ca. 0.896 cal g^-1^ ^o^C^-1^ referred to 1.000 cal g^-1^ ^o^C^-1^ of the DI water. In addition, the difference of heat flow between sAuNT water and DI water becomes more clearly at higher temperatures. These results suggest that most of adding energy in heating for DI water is used to maintain its degree of freedom in water molecules, preventing it from re-bonding of hydrogen bonds. Furthermore, the difference of heat capacity demonstrates that the sAuNT water is free and can present in the bulk DI water. These results are in agreement with the correlation of water cluster size and heat capacity, in which the heat capacity of (H_2_O)_21_ is smaller than that of (H_2_O)_50_ at 27 ^o^C^27^.

8. Excluding the contribution of impurity to sAuNT water with distinct properties

In this section, the influences of impurity to zeta potential, dissolved oxygen and specific heat are performed for demonstrating the intrinsic properties of sAuNT water. In addition to DI water and sAuNT water, the zeta potential of controlled water which is produced similarly to the sAuNT water but without illuminating by lamp or LED (produced in dark room) is measured and is -2.53 ± 0.49 mV. This value is almost same to -2.41 ± 0.31 mV of DI water and is obviously different to 31.2±0.70 mV of sAuNT water. Basally, the dissolved species from column for controlled water and sAuNT water should be same. However, the value of zeta potential is changed distinctly under LED illumination. It reveals the effect of the changed properties is dominated by the excited AuNPs, not the dissolved species.

In addition, according to the result of ICP-MS, sAuNT water contains about 0.05 ppb of Au atom perhaps from AuNPs or Au^3+^ salt. To exclude the effect factor, the experiments of dissolved oxygen and specific heat with various concentrations of AuNPs (10 and 50 ppb) and Au^3+^ salt (10 and 50 ppb) are examined. The previous study has reported that the dissolved oxygen (DO) of sAuNT water increases about 36.5 % to the DI water.^9^ Comparing with DI water, DO of DI water containing 10 ppb and 50 ppb of AuNPs do not change clearly. However, DO of DI water containing 10 ppb and 50 ppb of Au^3+^ salt decrease and decrease with the increase of concentration of Au^3+^ salt. It means the presence of Au^3+^ salt hinders oxygen dissolution. This result is contrary to that of sAuNT water, indicating that the enhancement of DO is irrelevant to the dissolved impurity.

Furthermore, the average specific heat of DI water containing 10 ppb of AuNPs, 50 ppb of AuNPs, 10 ppb of Au^3+^ salt and 50 ppb of Au^3+^ salt from 25 and 40 °C is 0.998, 0.997, 0.998 and 0.995 cal g^-1^ °C^-1^, respectively. These values are much close to 1.000 cal g^-1^ °C^-1^ of DI water and are totally different to 0.945 cal g^-1^ °C^-1^ of sAuNT water, indicating that the small amount of AuNPs or Au^3+^ salt existing in DI water do not affect the specific heat of water.

The above experiments demonstrate definitely the particular properties of sAuNT water are not contributed to the present impurities.


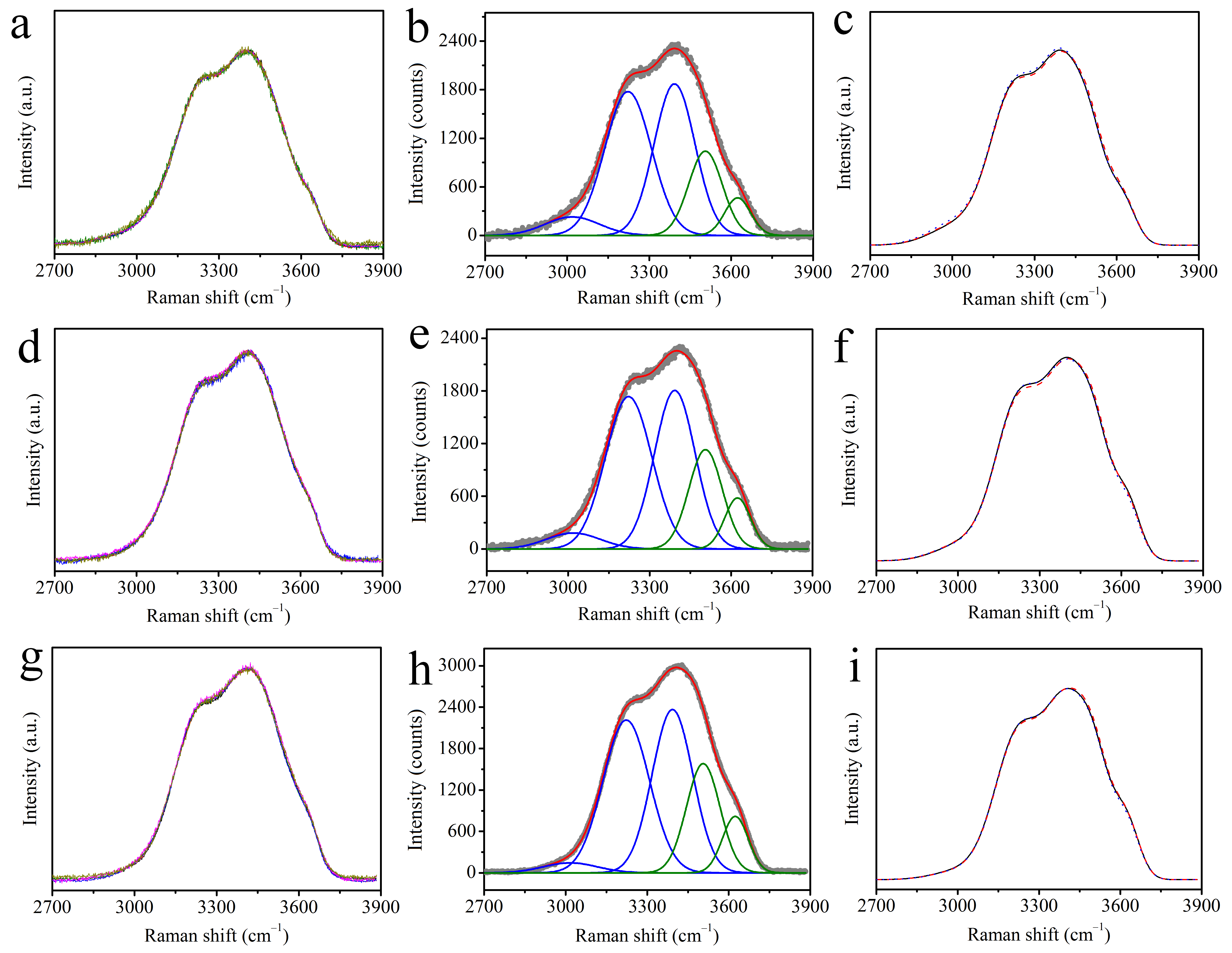


**Figure S1.** Raman spectra of O-H stretching vibration for waters. (a), (d) and (g) are Raman spectra of DI, AuNT and sAuNT waters (six samples) measured in different batches. The spectrum shapes do not change obviously, indicating that the measurement on O-H stretching vibration for waters based on Raman spectrum is reliable. (b), (e) and (h) are deconvoluted Raman spectrum of O-H stretching vibration. Generally, the Raman spectrum can be further deconvoluted into five component bands. Three component bands at lower frequency sides are associated with strong HB (blue lines); while two component bands at higher frequency sides are associated with weak/non- HB (green lines). The degree of non-hydrogen-bonded structure (DNHBS) for DI, AuNT and sAuNT waters, which are calculated on the area ratio of weak/non- HB to total HB, is 21.5±0.06 %, 24.5±0.15 % and 26.4±0.19 %, respectively. (c), (f) and (I) are the deviated deconvoluted spectra (blue and red lines) from optimally deconvoluted spectrum (black line) under extreme fitting conditions. The obtained values of DNHBS range from 20.01 to 22.70%, 24.23 to 25.07 % and 25.91 to 27.07 %, respectively.


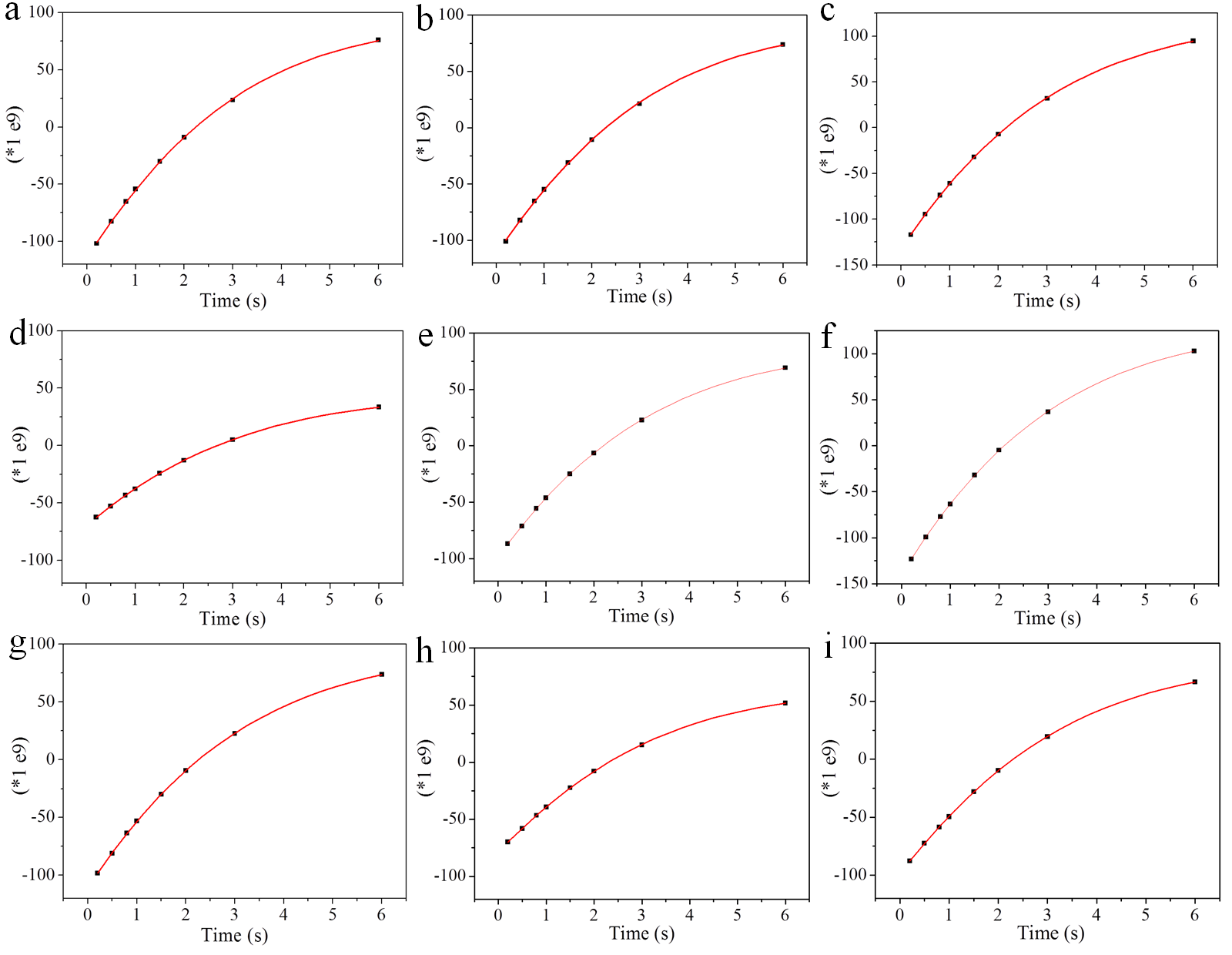


**Figure S2.** The spin-lattice relaxation time, *T*_1_, represents the time required for the longitudinal component of magnetization to recover its equilibrium value after applying a perturbing pulse sequence. It depends on the hydrogen-bonding interactions within water molecules. Spectra (a) and (b) represent plus of spectral signals as a function of repetition time for fresh DI water and sAuNT water, respectively. Spectra (c), (d) and (e) represent signals for aged DI water (NMR experiment performed in its fresh state), sAuNT water (without performing NMR experiment in its fresh state) and sAuNT water (NMR experiment performed in its fresh state), respectively, after their preparations for 10 days. Spectra (f) and (g) represent signals for fresh DI water and sAuNT water, respectively. Spectra (h) and (i) represent signals for aged sAuNT water (without placing on magnet) and sAuNT water (placing on magnet), respectively, after their preparations for 14 days.

**
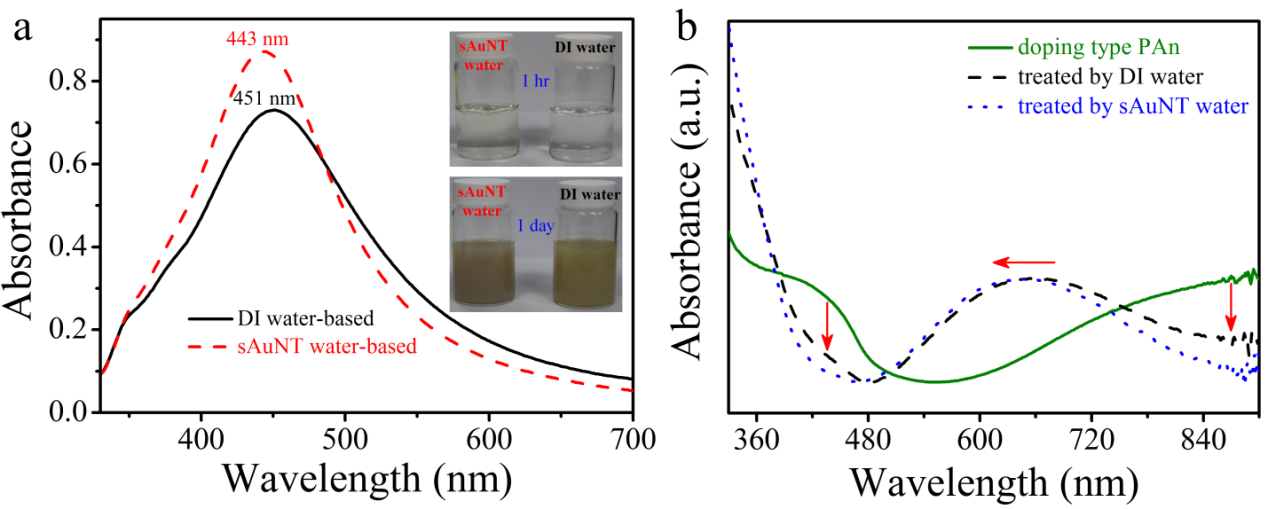
**

**Figure S3.** Reducing abilities of sAuNT water. (a) The absorption spectra of prepared silver nanoparticles (AgNPs) based on reductions of AgNO_3_ in deionized (DI) water and sAuNT water. The insert photos show yellow AgNPs-containing solutions after preparations for 1 h and 1 day. (b) Absorption spectra of doped PAn film deposited on conductive glass and its post-treatment with DI water and sAuNT water.
